# Automatic BASED scoring on scalp EEG in children with infantile spasms using convolutional neural network

**DOI:** 10.3389/fmolb.2022.931688

**Published:** 2022-08-10

**Authors:** Yuying Fan, Duo Chen, Hua Wang, Yijie Pan, Xueping Peng, Xueyan Liu, Yunhui Liu

**Affiliations:** ^1^ Department of Pediatrics, Shengjing Hospital of China Medical University, Shenyang, China; ^2^ School of Artificial Intelligence and Information Technology, Nanjing University of Chinese Medicine, Nanjing, China; ^3^ Department of Computer Science and Technology, School of Information Science and Technology, Tsinghua University, Beijing, China; ^4^ Ningbo Institute of Information Technology Application, CAS, Ningbo, China; ^5^ Australian AI Institute, FEIT, University of Technology Sydney, Sydney, NSW, Australia; ^6^ Department of Neurosurgery, Shengjing Hospital of China Medical University, Shenyang, China

**Keywords:** BASED score, scalp EEG, infantile spasms, convolutional neural network, deep learning

## Abstract

In recent years, the Burden of Amplitudes and Epileptiform Discharges (BASED) score has been used as a reliable, accurate, and feasible electroencephalogram (EEG) grading scale for infantile spasms. However, manual EEG annotation is, in general, very time-consuming, and BASED scoring is no exception. Convolutional neural networks (CNNs) have proven their great potential in many EEG classification problems. However, very few research studies have focused on the use of CNNs for BASED scoring, a challenging but vital task in the diagnosis and treatment of infantile spasms. This study proposes an automatic BASED scoring framework using EEG and a deep CNN. The feasibility of using CNN for automatic BASED scoring was investigated in 36 patients with infantile spasms by annotating their long-term EEG data with four levels of the BASED score (scores 5, 4, 3, and ≤2). In the validation set, the accuracy was 96.9% by applying a multi-layer CNN to classify the EEG data as a 4-label problem. The extensive experiments have demonstrated that our proposed approach offers high accuracy and, hence, is an important step toward an automatic BASED scoring algorithm. To the best of our knowledge, this is the first attempt to use a CNN to construct a BASED-based scoring model.

## 1 Introduction

Infantile spasms (IS) is one of the most common age-dependent epileptic encephalopathies in infancy, most of which are difficult to treat and have a poor prognosis (D’Alonzo et al., 2018; Kesavan and Sankhyan, 2019). Also known as the WEST syndrome, IS is characterized by a triad of symptoms: epileptic spasm, which is the major seizure type in infancy; impairment of psychomotor development; and the presence of hypsarrhythmia in the interictal electroencephalogram (EEG) (Wilmshurst et al., 2015). The approximate incidence rate of IS in infants is 0.2–0.5 % with geographical differences, and the peak age of onset is 4–7 months (D’Alonzo et al., 2018; Jia et al., 2018). IS should be diagnosed and treated as early as possible, especially in patients with predictors of poor outcome (Bitton et al., 2021; Kvernadze et al., 2021). However, only 29% of children with IS achieve diagnosis and treatment within 1 week of onset, mainly due to the neglect of epileptic spasms and misreading EEG changes (Hussain et al., 2017; Harini et al., 2020).

The EEG features of IS are complex and show a variety of characteristics. Hypsarrhythmia is the typical feature of EEG during the interictal period and is an important diagnostic criterion and index for therapeutic evaluation in IS. However, many variant forms of hypsarrhythmia in IS exist, with an occurrence rate of up to 69%) (Demarest et al., 2017; Nenadovic et al., 2018). It has been shown that different EEG evaluators have poor inter-rater reliability (IRR) in the assessment of typical hypsarrhythmia and other atypical EEG features of IS (Hussain et al., 2015). It has further been found that quantitating characteristics of hypsarrhythmia improves the accuracy of EEG diagnosis for IS, leading to earlier treatment initiation (Smith et al., 2018). Therefore, it is necessary to raise the reliability and validity of EEG evaluation methods for IS, such as EEG grading scales, and to improve the IRR of these methods.

At present, several EEG grading scales for IS have been developed. Among them is the Burden of Amplitudes and Epileptiform Discharges (BASED) score, which is a novel and simplified EEG grading scale, and the most used. This scoring method uses a 6-point scale based on the interictal EEG of IS, with 0 being normal and 5 being the most epileptic (Mytinger et al., 2015). The 2015 version of the BASED score was the first reported version (Mytinger et al., 2015). After modification and further validation, the 2021 version was published (Mytinger et al., 2021). The BASED score has been widely used as a criterion for the evaluation of EEG in pre-treatment and post-treatment studies of patients with IS (Wang et al., 2021; Yan et al., 2021; Wan et al., 2022). The definition of remission is that, for a pre-treatment score of 4 or 5, the score must improve to 3 or less; and for a pre-treatment score of 3, the score must improve to 2 or less. Although the BASED score has shown excellent IRR, relying only on a manual process and human visual recognition to quantify EEG data remains subjective and challenging. Manual EEG annotation is highly time-consuming, and BASED scoring is no different. Therefore, it is important to establish a standard, automatic EEG scoring system that uses intelligent quantification of EEG data to achieve time-savings while maintaining high reliability and validity. This will assist the clinical diagnosis of IS and its monitoring during treatment.

In the past few decades, convolutional neural networks (CNNs) have shown great potential in multiple fields, such as computer vision and speech recognition (LeCun et al., 2015). This method is suitably scaled for large datasets, as a hierarchical structure in natural signals can be exploited. As such, CNNs appear suitable for analyzing long-term, multi-dimensional, and highly non-stationary signals, such as EEG signals. Several studies have explored the application of CNNs to EEG analysis in healthy populations in areas such as sensory processing, cognitive-emotional processing, speech, and motor planning/execution and have achieved excellent performance (Sussillo et al., 2016; Schirrmeister et al., 2017; Lawhern et al., 2018).

CNNs are proven to have huge potential for EEG classification problems. However, studies focusing on using CNN for BASED scoring, a challenging but vital task in the diagnosis and treatment of IS, are few. Motivated by this fact, we here propose a multi-layer CNN for automatic BASED scoring. We tested our model with the long-term EEG recordings of 11 patients with IS, annotating them into four levels of the BASED score for clinical use. The results demonstrated an excellent classification accuracy of 96.9% on the validation set. To the best of our knowledge, this is the first attempt to use a CNN to construct a BASED scoring algorithm.

## 2 Materials and methods

### 2.1 Subjects

We retrospectively included patients meeting the diagnosis standard of IS from 2019 to 2021 at Shengjing Hospital of China Medical University. All patients with IS also met the following inclusion criteria: 1) each patient with two scalp video-EEG data available, the diagnostic EEG before treatment and the first video-EEG after treatment; 2) patients with 4 h of video-EEG monitoring, including a slow-wave sleeping period; 3) patients aged between 3 and 12 months during the video-EEG examination; and 4) patients with generalized epileptic spasms. The study was approved by the Research Ethics Board of Shengjing Hospital of China Medical University with IRB number 2022PS692K.

### 2.2 Data acquisition

Using the standard international 10–20 system with 16 channels, EEG was recorded at a sampling rate of 1,000 Hz with a video–EEG system (Nihon Kohden) and a low-cut frequency (LCF) filter of 0.5 Hz was used. The 2021 BASED score was used to quantify the EEG changes of IS (Mytinger et al., 2021). As this standard required, the most epileptic, 5-min clean EEG data during interictal slow-wave sleep was chosen in each patient by two expert EEG reviewers (Mytinger et al., 2021). All EEG clips were then shuffled into a random order, with all personal and identifying information completely removed. Another three expert EEG reviewers were blinded and scored the EEGs randomly. According to the BASED score system, all the EEG clips were divided into four levels as BASED scores 5, 4, 3, and ≤2. The datasets are available from the corresponding author upon reasonable request.

### 2.3 Data preprocessing

**FIGURE 1:**
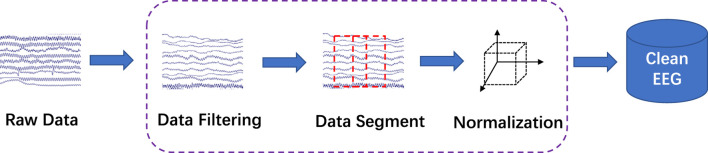
EEG preprocessing. The raw EEG was band-pass filtered at [0.5*Hz*, 128*Hz*]. A 5-s sliding window with a 2-s overlap was used to crop the long-term EEG into segments.

Two epileptologists manually read the long-term EEG and annotated the BASED score. A 64-order butter-worth band-pass filter ([0.5*Hz*, 128*Hz*]) was enabled to eliminate the noises. The EEG was then cropped into segments using a 5-s sliding window with a 2-s overlap, and then a z-score method was used to normalize the EEG data. Figure 1 illustrates the preprocessing.

Given a multi-channel EEG dataset *X* with patient *i* ∈ {1, 2, … , *N*}, where *N* is the number of patients. Each dataset was divided into segments, as described earlier. Concretely, given dataset $D^{i}=\left(X^{1},y^{1}\right),\left(X^{2},y^{2}\right),\ldots ,\left(X^{N_{i}},y^{N_{i}}\right)$ , where *N*_*i*_ denotes the total number of segments for patient *i*. The *j*-th EEG segment *X* ^*j*^ ∈ **R** ^*C*.*T*^, 1 ≤ *j* ≤ *N*_*i*_ contains *C* channels and *T* time points per segment, where *C* = 16 and *T* = 5*1,000 = 5000, in this study. The class label of segment *j* is denoted by *y* ^*j*^ ∈ { ≤ 2, 3, 4, 5}, corresponding to the BASED score from 0 to 5.

### 2.4 Problem formulation

The EEG segments were divided under a 5-fold cross-validation strategy. Since the EEG were manually annotated into four levels based on the BASED scores, the experimental task here was a 4-label classification problem.

### 2.5 Convolutional neural network

**FIGURE 2:**
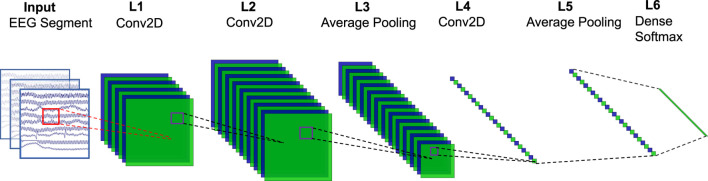
Network structure. The input is a multi-channel EEG segment with a dimension of 16 (*channel*) × 5000 (*sampling point*, 5 × 1,000). L1 is a temporal filter that contains 8 filters with a 1 × 32 kernel. L2 is a spatial filter that contains 16 filters with a 16 × 1 kernel. In L3, the pool size is (1, 3), and the stride is none. L4 contains 16 filters with 1 × 16 kernels. In L5, the pool size is (1, 8), and the stride is none. L6 is a dense layer with softmax activation.

In this study, the proposed BASED scoring algorithm was made of a CNN. The multi-layer CNN model structure is illustrated in Figure 2. Our model consisted of three convolutional layers with 8, 16, and 16 filters. A batch normalization layer was performed after each convolutional layer. The convolutional layers were followed by two average-pooling layers. An activation layer was performed before each average-pooling layer. Meanwhile, a dropout layer was followed by each average-pooling layer. A dense layer with softmax activation was used as a classification output layer, which divided the EEG segments into four levels. The network referred to previous research works to use CNN in EEG decoding, including, DeepConvNet, ShallowConvNet (Schirrmeister et al., 2017), and EEGNet (Lawhern et al., 2018).

The input to the CNN structure was the band-pass filtered 16-channel scalp EEG. A CNN predictor was trained to assign the input segment *X* ^*j*^ a class label, i.e., *f* (*X* ^*j*^; *θ*) ∈ **R** ^*C*.*T*^ → **R** ^*P*^, where *θ* was the parameter set of CNN, *C* = 16 was the number of channels, *T* = 1,000, ×, 5 = 5000 were the time points, and *P* ∈ { ≤ 2, 3, 4, 5} was the possible output label. The model contains three convolutional layers, three batch normalization layers, two average-pooling layers, and a dense layer with softmax activation. The first two convolutional layers corresponded to the temporal filtering and spatial filtering of the EEG segment. In the first layer, each filter performed a convolution over time, while in the second layer, each filter performed spatial filtering with weights for all possible pairs of electrodes with filters of the preceding temporal convolution. After each average-pooling layer, a 50% dropout was used to avoid over-fitting.

### 2.6 Experiment setup

#### 2.6.1 Model evaluation and implementation

We conducted a comprehensive evaluation in this study by using the proposed model to classify the long-term EEG into four levels of BASED scores. A *5*-fold cross-validation was designed in our experiments. The whole gold standard dataset was divided into five portions. In each repeated iteration, we randomly used one portion of the data as testing data and applied the rest four portions of the data as training data. This process would be repeated *5* times until all data had been tested once. The classification performance was evaluated by aggregating all iterations. Our approach was implemented with Tensorflow 2.3.0. For training models, we used Adam with a batch of 16 EEG segments and 300 epochs. The drop-out rate was 0.5 for our approach.

#### 2.6.2 Evaluation metrics

We calculated true positive (TP), false positive (FP), true negative (TN), and false negative (FN) for the classification by comparing the classified labels and gold-standard labels. Then, we calculated the accuracy, precision, and recall as follows:

$$\begin{array}{ll} & Accuracy=\frac{TP+TN}{TP+TN+FP+FN}\\ & Precision=\frac{TP}{TP+FP}\\ & Recall=\frac{TP}{TP+FN}. \end{array}$$ (1)

## 3 Results and discussion

### 3.1 Sample

A total of 36 patients (26 boys and 10 girls) with IS were finally included (age range, 3–12 months; mean age, 6.58 months; standard deviation, 2.46). A total of 72 4-h video-EEG datasets were obtained for testing. The detailed information is shown in Table 1.

**TABLE 1:** BASED score for EEG clips

	BASED score			
	5	4	3	≤2
EEG clip number	21	25	15	11
Age (months) (M± SD)	6.57 ± 2.56	7.00 ± 2.69	6.13 ± 1.99	6.09 ± 2.26

### 3.2 Performance evaluation

To verify the classification performance of our proposed model, we tested it on both the validation set and training set. Table 2 shows the classification performance of our model for evaluating BASED score on the training set and validation set. By adopting a 5-fold cross-validation strategy in the validation set, our model achieved a mean accuracy of 96.9%, which is highly accurate in evaluating the BASED score automatically. Meanwhile, our model achieved similar performance on the training set, which was a mean accuracy of 95.9% in evaluating the BASED score. Table 2 shows the similar superior performance on recall and precision, which reflected the proportion of true positive. Also, it is of note that our model brought a suitable trade-off on annotating EEG segments into four levels of BASED scores.

**TABLE 2:** Training and validation set performance over 300 epochs for our model.

Dataset	Accuracy (%)	Precision (%)	Recall (%)
Training set	95.9 ± 0.26	91.9 ± 0.33	91.6 ± 0.25
Validation set	96.9 ± 0.36	93.9 ± 0.17	93.8 ± 0.24

### 3.3 Multi-label classification performance evaluation

**FIGURE 3:**
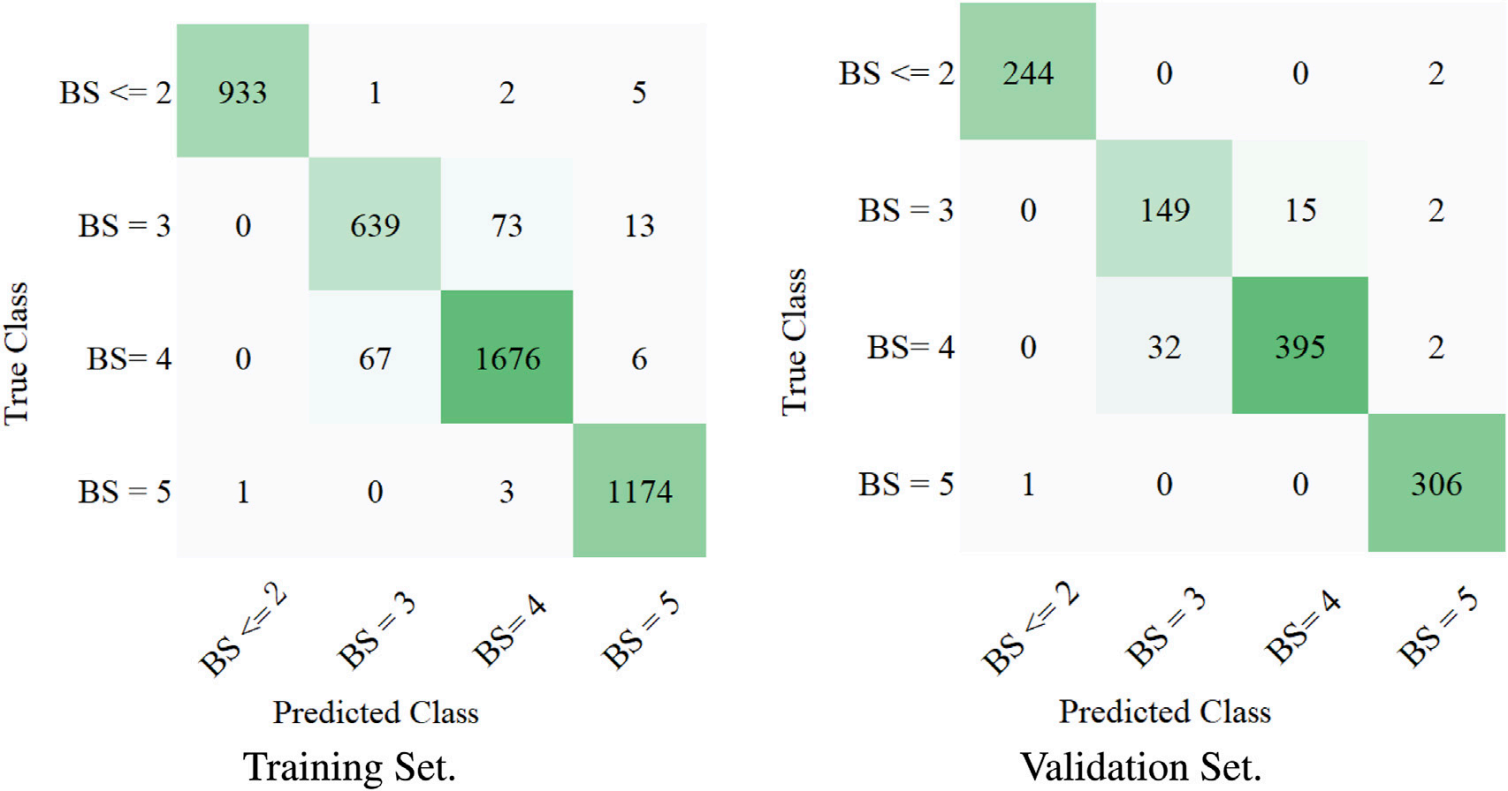
Confusion matrix for the training set and validation set. BS represents the BASED score.

To measure the multi-label classification performance of our model, we introduced a confusion matrix as a metric. Figure 3 shows the full confusion matrix of the training set and validation set. Some misclassifications are presented in Figure 3, but the overall classification performance of our model was highly accurate. This was quantified in Table 3 *via* precision and recall. For four levels of BASED scores, the average precision was over 88%. Table 3 shows a similar multi-label classification performance on recall, which quantified the number of correct positive predictions made out of all positive predictions. Dividing EEG segments into four levels of BASED score benefited from the convolutional layers for highly accurate classification. In contrast, from Figure 3 and Table 3, it is clear that only a few EEG segments were classified in the wrong category on the validation set and training set.

**TABLE 3:** Accuracy, precision, and recall in each class. BS represents the BASED score.

Class	Accuracy (%)	Precision (%)	Recall (%)
BS ≤2 (training set)	99.80	99.15	99.19
BS = 3 (training set)	96.65	88.14	90.38
BS = 4 (training set)	96.71	95.83	95.55
BS = 5 (training set)	99.39	99.66	98.00
BS ≤2 (validation set)	99.74	99.19	99.59
BS = 3 (validation set)	95.73	89.76	82.32
BS = 4 (validation set)	95.73	92.07	96.34
BS = 5 (validation set)	99.39	99.67	98.08

### 3.4 Performance of the proposed model with 5-fold cross-validation

**FIGURE 4:**
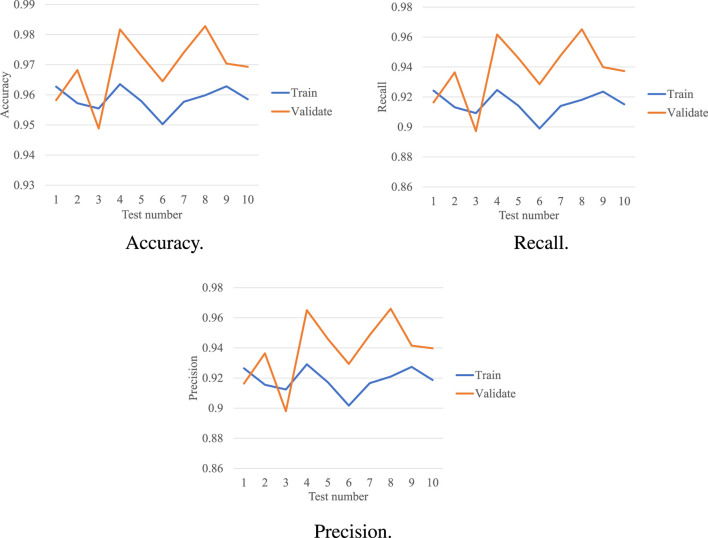
Performance of our model in the 5-fold cross-validation.

The performance of our model in the 5-fold cross-validation test is illustrated in Figure 4. As shown, we repeated the experiment 10× and averaged the results. In the validation set, the accuracy of each test was much higher than the chance level (1/4 = 25%). The highest accuracy was 98.2%, while the lowest accuracy was 94.9%. A consistent accuracy of over 94% was obtained across all the tests. The average accuracy was 96.9%, which was also very promising. In the training set, the highest accuracy was 96.4%, while the lowest accuracy was 95.1%. An accuracy of >95% was consistently obtained across all tests. The average accuracy was 95.9%.

### 3.5 Discussion

Our motivation was to investigate the performance of a CNN, which is known to have great potential in clinical research and solutions, in BASED scoring for IS. As indicated in the previous sections, the scalp EEG was used as the input of a multi-layer CNN pain detector. While classifying the EEG segments under 4-levels BASED score, a consistent accuracy of over 90.0% was obtained across all classes. The average accuracy was above 95.0%, which was very promising. BS = 3 was the only class where precision and/or recall was lower than 90% on either the training or validation set. The recall of BS = 3 was 82.32%, which was the lowest across all the classes on the validation set. This indicates that BS = 3 is more likely to be misclassified when compared with the other classes.

The network structure used here was in reference to previous research works to use CNN in EEG decoding, including, DeepConvNet, ShallowConvNet (Schirrmeister et al., 2017), and EEGNet (Lawhern et al., 2018). The results proved that the CNN-based model can achieve very high accuracy for BASED scoring. In general, the proposed method showed outstanding performance, obtaining high accuracy in the 4-level classification problem, proving its good stability to handle the BASED scoring.

Although our study produced favorable findings, there are still some limitations. The raw EEG data used in our study only came from 3–12 months old patients with generalized epileptic spasms. It requires to be verified whether the proposed method can be applied to all the EEG datasets of IS with higher age and/or with focal epileptic spasms. The sample size of our study was small, and the clinical application of the proposed method requires future confirmation in large-sample clinical trials. It is worth mentioning that the CNN network contains many hyperparameters, e.g., the number and size of the kernels in each convolution layer, the size of the stride, and the size of the kernels in the pooling layer. Although the proposed method achieved high computational accuracy, its computational cost must be considered for further optimization.

## 4 Conclusion

Recent advances in neuroimaging techniques and machine learning algorithms have significantly enhanced ongoing research works on automatic EEG analysis. Our study investigated the usability of a CNN in BASED scoring. The problem was formulated into a 4-label classification problem (Score 5, 4, 3, and ≤2) according to the BASED score. Using the proposed multi-layer network model, a remarkable classification accuracy of above 96% was achieved in the validation set. Further research will focus on the interpretability of the layers and the optimization of the model structure.

## Data Availability

The raw data supporting the conclusions of this article will be made available by the authors, without undue reservation.
